# Sonic Hedgehog Gene Delivery to the Rodent Heart Promotes Angiogenesis via iNOS/Netrin-1/PKC Pathway

**DOI:** 10.1371/journal.pone.0008576

**Published:** 2010-01-05

**Authors:** Rafeeq P. H. Ahmed, Khawaja Husnain Haider, Jiang Shujia, Muhammad Rizwan Afzal, Muhammad Ashraf

**Affiliations:** Department of Pathology and Laboratory Medicine, University of Cincinnati, Cincinnati, Ohio, United States of America; Harvard Medical School, United States of America

## Abstract

**Background:**

We hypothesized that genetic modification of mesenchymal stem cells (MSCs) with Sonic Hedgehog (Shh) transgene, a morphogen during embryonic development and embryonic and adult stem cell growth, improved their survival and angiogenic potential in the ischemic heart via iNOS/netrin/PKC pathway.

**Methods/Principal Findings:**

MSCs from young Fisher-344 rat bone marrow were purified and transfected with pCMV Shh plasmid (^Shh^MSCs). Immunofluorescence, RT-PCR and Western blotting showed higher expression of Shh in ^Shh^MSCs which also led to increased expression of angiogenic and pro-survival growth factors in ^Shh^MSCs. Significantly improved migration and tube formation was seen in ^Shh^MSCs as compared to empty vector transfected MSCs (^Emp^MSCs). Significant upregulation of netrin-1 and iNOS was observed in ^Shh^MSCs in PI3K independent but PKC dependent manner. For *in vivo* studies, acute myocardial infarction model was developed in Fisher-344 rats. The animals were grouped to receive 70 µl basal DMEM without cells (group-1) or containing 1×10^6^ ^Emp^MSCs (group-2) and ^Shh^MSCs (group-3). Group-4 received recombinant netrin-1 protein injection into the infarcted heart. FISH and *sry-*quantification revealed improved survival of ^Shh^MSCs post engraftment. Histological studies combined with fluorescent microspheres showed increased density of functionally competent blood vessels in group-3 and group-4. Echocardiography showed significantly preserved heart function indices post engraftment with ^Shh^MSCs in group-3 animals.

**Conclusions/Significance:**

Reprogramming of stem cells with Shh maximizes their survival and angiogenic potential in the heart via iNOS/netrin-1/PKC signaling.

## Introduction

Sonic hedgehog (Shh) gene is one of the highly conserved mammalian hedgehog genes and has a wide distribution in a variety of tissues during embryonic development [1]. The post-natal intrinsic activity of Shh remains intact albeit with sub-optimal functioning and is reactivated under tissue ischemia and in various pathologies, including tumors [2], [3]. Such post-natal reactivation of the embryonic signaling pathways implying Shh in response to muscle injury in the animal models incurred enhanced angiomyogenic response [4], [5]. In one of the experimental studies, more than 15-fold increase in Shh mRNA expression was observed in the ischemic myocardium [6]. An outside intervention to overexpress Shh in the heart activated its downstream signaling cascade and strongly induced Patched1 (Ptc1) expression in the cardiomyocytes which indicated an active participation of Shh in the myocardial repair process. Interestingly, activation of Shh signaling caused upregulation of pro-angiogenic growth factors including vascular endothelial growth factor (VEGF) and angiopoietin-1 which resulted in an increased angiogenic response and globally improved the heart function.

Bone marrow derived stem cells (BMSCs) which have been shown to improve heart function, attenuate infarct size expansion and contribute to myocardial regeneration both in the experimental as well as in clinical settings [7]–[10]. In addition, BMSCs are excellent carriers of therapeutic genes to the heart [11]–[13]. In the present study we took advantage of the anti-apoptotic and pro-angiogenic role of Shh signaling and combined Shh transgene delivery to the infarcted heart by transplantation of mesenchymal stem cells (MSCs) which were non-virally transfected to overexpress Shh. The anticipated objective of our multipronged strategy was to achieve intracrine, autocrine, and paracrine effects of Shh protein which was secreted from MSCs overexpressing Shh (^Shh^MSCs) and regenerated the damaged tissue, induced revascularization and concomitantly prevented remodeling of the heart by preserving the existing myocardium.

Treatment of the cells with Shh or instrinsic Shh gene overexpression in response to various factors involve signaling pathways including PI3K/Akt, Ras/ERK and PKC. PKC participates as a mediator of signal transduction during multiple cellular responses to cellular signals which stimulate cell proliferation, and differentiation. Our results showed that Shh transgene over expression in MSCs initiated PKC signaling which was characterized by the activation of PKM, a catalytic fragment of PKC. ^Shh^MSCs showed better survival post-engraftment as compared with the empty vector transfected control MSCs (^Emp^MSCs). Moreover, we report that Shh upregulated angiogenic genes such as netrin-1, iNOS, VEGF and angiopoietins, which play a significant role in Shh induced angiogenesis. Shh-induced upregulation of netrin-1 and iNOS was PKC dependent. We further observed that PKM was upregulated in ^Shh^MSCs and was sensitive to cyclopamine and chel pretreatment of the cells.

## Materials and Methods

Detailed methods are available in Text S1. Table S1 shows the antibodies used for immunohistology and Western blotting, and Table S2 shows the primers used for RT-PCR. Young female Fischer-344 rats (n = 30) each weighing 180–200 g were used in this study. The present study conformed to the Guide for the Care and Use of Laboratory Animals published by the US National Institutes of Health (NIH Publication No. 85-23, revised 1996) and protocol approved by the Institutional Animal Care and Use Committee, University of Cincinnati. All surgical manipulations were carried out under general anesthesia.

## Results

### *In vitro* Studies

**Figure 1:**
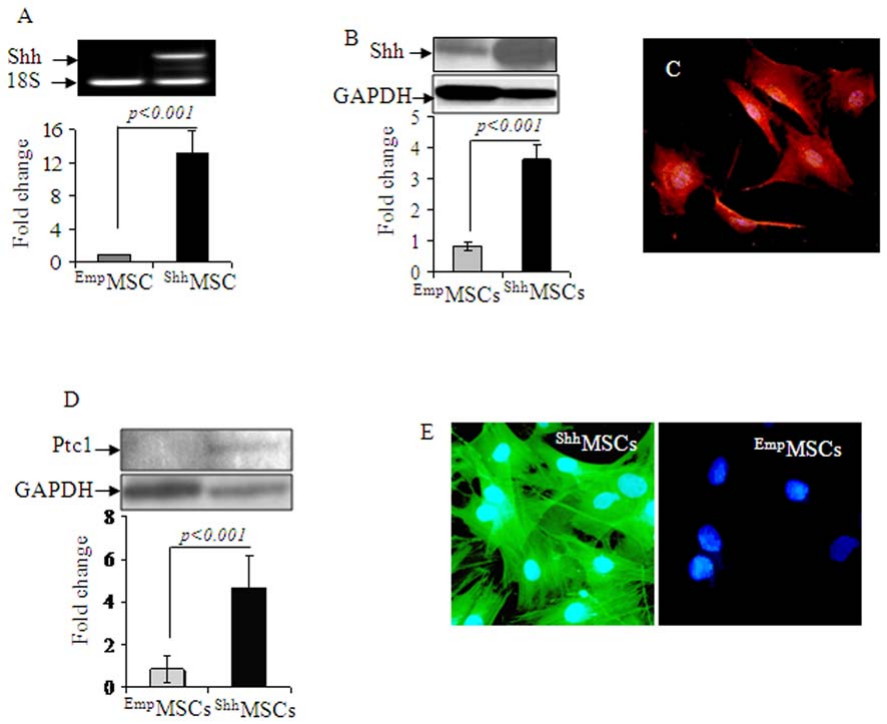
In vitro characterization of ^Shh^MSCs. (A) RT-PCR and (B) Western blotting showed significant amplification of Shh transgene and Shh protein expression in ^Shh^MSCs as compared with ^Emp^MSCs on 72-h after transfection. (C) Fluorescent immunostaining of ^Shh^MSCs for Shh overexpression (red fluorescence) at 72-h after trasnfection of Shh plasmid (magnification = 100x). (D) Western blot and (E) Fluorescent immunostaining revealed elevated expression of Ptc-1 in ^Shh^MSCs (green fluorescence) as compared with ^Emp^MSCs (magnification = 100x).

Shh plasmid was successfully constructed using commercially available pCMV Script Vector using mRNA isolated from 14-day rat embryo and the plasmid construct was sequenced for correct insertion of Shh transgene (Figure S1A & Figure S1B). RT-PCR and Western blotting revealed significantly elevated expression of Shh gene (12-fold) and protein (4.5-fold) in ^Shh^MSCs as compared with ^Emp^MSCs (Figure 1A & 1B). Fluorescent immunostaining showed that more than 65% cells stained positive for Shh transgene overexpression (Figure 1C). At 72-h after transfection with Shh, Ptc1 gene expression was 2.7-fold higher in ^Shh^MSCs as compared with ^Emp^MSCs. These findings were confirmed by Western blot (Figure 1D) and fluorescent immunostaining (Figure 1E). Flow cytometry showed that Shh overexpression did not alter surface marker expression in ^Shh^MSCs (Figure S1C).

### Shh Induced Angiogenic Growth Factor Expression in MSCs

**Figure 2:**
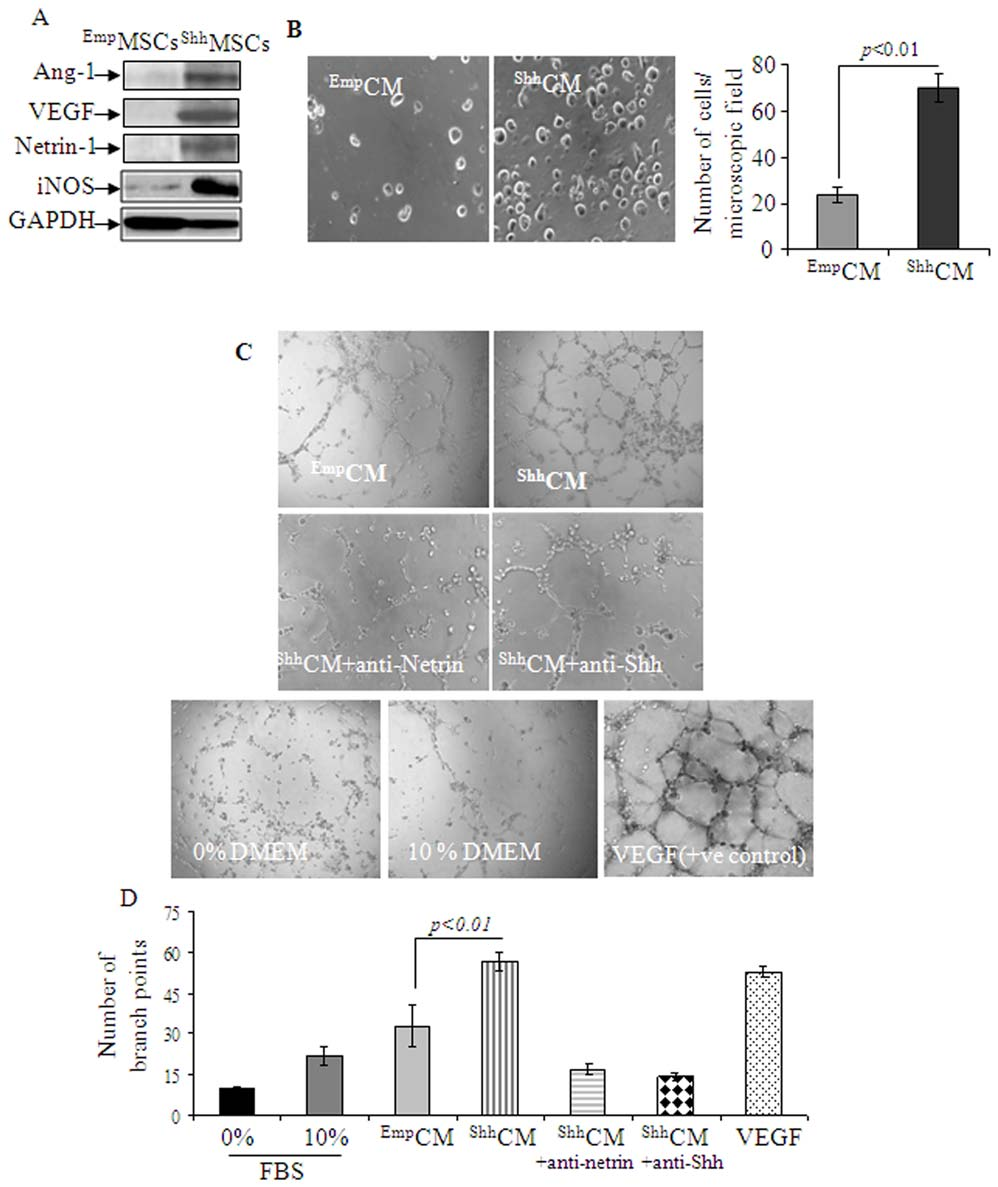
Expression of secretable angio-competent growth factors from ^Shh^MSCs. A. Shh overexpression in MSCs induced significant overexpression of multiple factors including Ang-1, VEGF, netrin-1 and iNOS as compared with ^Emp^MSCs. (B) Invasion assay showing significantly higher migration of HUVECs in response to ^Shh^CM as compared with ^Emp^CM. (C) Culture of HUVECs in ^Shh^CM promoted their differentiation into tubular network structures in vitro. Representative photomicrographs of HUVEC cultures for some experimental conditions as labeled in the individual photomicrograph (Magnification = 200x). (D) Quantitative analysis of network formation (formation of vascular assembly in culture) under the different experimental conditions expressed as network projections per low-power field. Results are the mean±SE from triplicate samples.

In addition to upregulation of Ptc1, overexpression of Shh in MSCs stimulated the expression of secretable angiogenic growth factors including Ang-1 and VEGF. Our custom-made real-time PCR based array of 72 genes revealed multiple angiogenic growth factors showing more than 2-fold increase as compared to ^Emp^MSCs (Table S3). Western blot studies using cell lysate protein samples from ^Shh^MSCs and ^Emp^MSCs confirmed these findings (Figure 2A).

### Shh Caused Endothelial Mobilization and Tube Formation

We investigated the ability of human umbilical vein endothelial cells (HUVECs) to migrate toward conditioned medium from ^Shh^MSCs (^Shh^CM) using a modified Boyden chamber assay (Figure 2B). Incubation of HUVECs with ^Shh^CM for 4-h stimulated increased chemotaxis of HUVECs in a Transwell system as compared with ^Emp^MSCs. In vitro angiogenic response of HUVECs to ^Shh^CM was determined by tube formation assay on matrigel which showed that morphological changes were most obvious at 6-h after incubation of the cells with ^Shh^CM as compared with conditioned medium from empty vector transfected MSCs (^Emp^CM) (Figure 2C). Quantification of the number of branch points per low power microscopic field showed that the number of tubular structures were higher in ^Shh^CM as compared to the cells treated with ^Emp^CM (*p*<0.01), and 10% FBS supplemented DMEM (*p*<0.001) (Figure 2D). The formation of tubular structures was abolished by prior treatment of the cells with anti-Shh (*p*<0.01 vs ^shh^CM without anti Shh antibody) and anti Netrin-1 antibodies (*p*<0.01 vs ^shh^CM without anti-Shh antibody). Basal DMEM without FBS supplementation failed to induce any morphological changes in terms of tube formation (Figure 2D).

### Shh Overexpression and Molecular Signaling in MSCs

**Figure 3:**
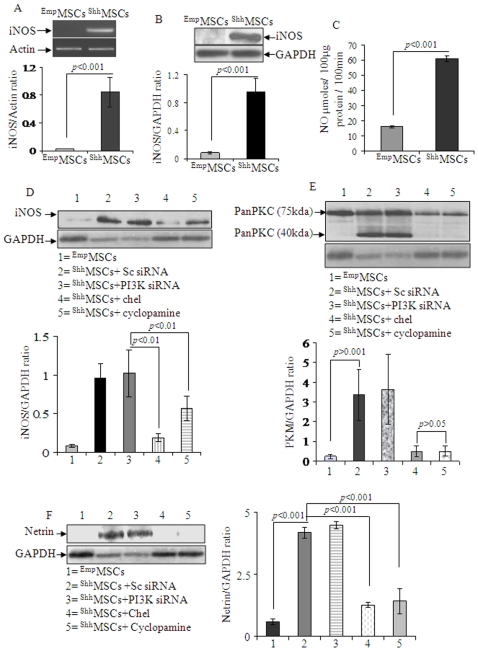
^Shh^MSCs upregulated iNOS and netrin-1 expression. (A) RT-PCR and (B) Western blot showing significantly higher level iNOS gene and protein expression respectively in ^Shh^MSCs as compared with ^Emp^MSCs. (C) iNOS activity assay showed increased NO production (in 100-min/100 µg protein) in ^Shh^MSCs as compared with ^Emp^MSCs. This result was in accordance with higher level expression of iNOS in ^Shh^MSCs thus indicating its functionally active status. (D) Transfection of ^Shh^MSCs with Sc siRNA and PI3K specific siRNA failed to abrogate iNOS expression. On the other hand, pretreatment of ^Shh^MSCs with 2.5 µM chel or 1 µM cyclopamine significantly blocked iNOS expression in ^Shh^MSCs. (E) Western blot and densitometry of changes in PKM expression in ^Shh^MSCs showed significantly higher level expression of 45 kDa fragment of PKC (PKM) in ^Shh^MSCs as compared to ^Emp^MSCs which was not blocked by PI3K specific RNA interference using Sc siRNA as a control. However, PKM fraction was sensitive to 2.5 µM chel or 1 µM cyclopamine. (F) Western blot showing significantly higher level protein expression of netrin-1 in ^Shh^MSCs (Lanes-2 & 3) as compared with ^Emp^MSCs (Lane-1). Netrin-1 expression was not abrogated by transfection of ^Shh^MSCs with PI3K siRNA or Sc siRNA (Lanes-2 & 3). However, netrin-1 expression in ^Shh^MSCs was abrogated by pretreatment of ^Shh^MSCs with 2.5 µM chel (Lane-4) or 1 µM cyclopamine (Lane-5).

iNOS gene expression was significantly increased in ^Shh^MSCs as compared to ^Emp^MSCs (Figure 3A) which was confirmed by Western blot (Figure 3B). Measurement of NO activity by using a colorimetric NO assay kit showed that Shh overexpression was associated with a concomitant increase in NO production in ^Shh^MSCs (Figure 3C). For every 100 µg protein, the amount of NO produced in 100-min was 15 µmoles in ^Emp^MSCs and was 60 µmoles for ^Shh^MSCs. These results showed that iNOS expression in ^Shh^MSCs was biologically active and contributed to the production of NO.

Next we investigated whether PI3-kinase/Akt pathway was involved in Shh-induced effects in MSCs. PI3K gene was successfully knocked down in MSCs by transfection with PI3K siRNA as compared to scrambled siRNA (Sc siRNA) transfected cells which was indicated by abrogation of pAkt expression (Figure S2). During Western blot studies, subsequent Shh transfection of the respective siRNA transfected cells showed that PI3K/Akt abrogation failed to block Shh induced iNOS expression (Figure 3D; lane-3). On the contrary, treatment of MSCs with 2.5 µM chel or 1 µM cyclopamine prior to Shh transfection significantly abolished iNOS expression in ^Shh^MSCs (Figure 3D). We also found that Shh overexpression also induced panPKC fragment PKM (45 kd; a proteolytic subunit of PKC) in ^Shh^MSCs which was completely abolished by pretreatment of the cells with 2.5 µM chel or 1 µM cyclopamine (Figure 3E). Prolonged activation of PKC can result in its proteolysis to the constitutively active catalytic fragment protein kinase-M, which would dissociate from the sarcolemma and phosphorylates proteins such as myosin that are inaccessible to membrane-bound protein kinase-C. PKM induces relaxation of smooth muscle fibers contracted at sub-maximal Ca^2+^ concentrations [14]. However, PI3K specific RNA interference failed to abolish PKM (Figure 3E).

### Shh Upregulated Netrin-1 and iNOS Expression

Real-time PCR showed more than 60-fold increase in netrin-1 mRNA levels (Table S3). Western blot showed higher level of netrin-1 in ^Shh^MSCs as compared to ^Emp^MSCs and similar to iNOS expression, pretreatment of ^Shh^MSCs with 2.5 µM chel and 1 µM cyclopamine significantly abolished netrin-1 expression (Figure 3F). These results indicated that PKC was essential for Shh mediated upregulation of both iNOS and netrin-1.

### ^Shh^CM Improved Cell Survival

Using release of LDH as an indicator of cellular injury, LDH release assay showed that treatment of native MSCs (Figure S3) and H9C2 cardiomyocytes (Figure S4) with ^Shh^CM was cytoprotective and prevented cell death under oxidant stress.

### *In vivo* Studies

**Figure 4:**
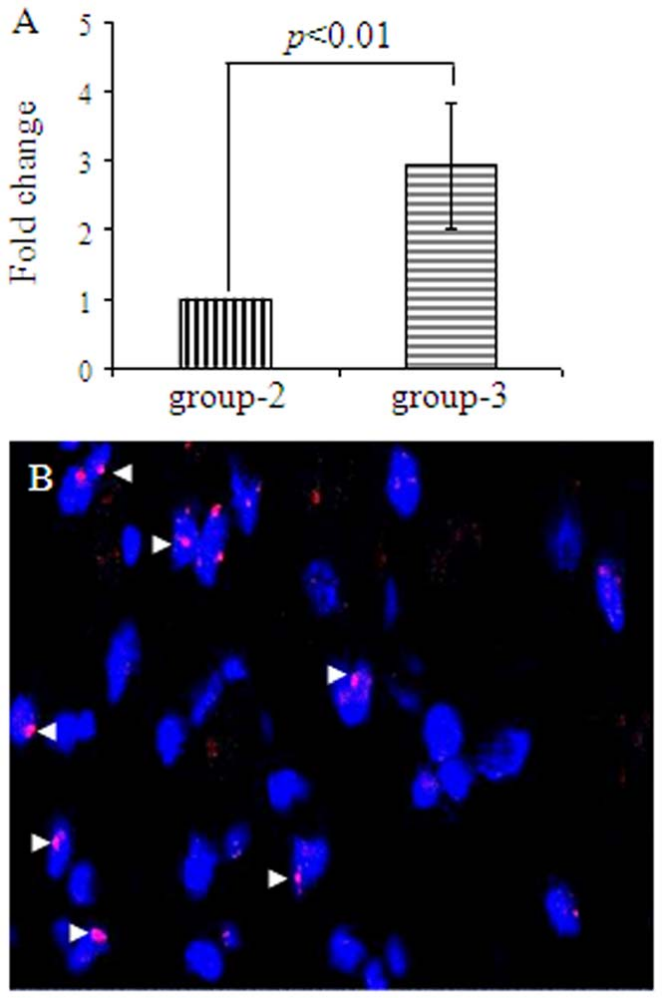
^Shh^MSCs survival in the infarcted heart. (A) Real-time PCR for *sry*-gene showed improved survival of ^Shh^MSCs in the infarcted rat heart on day-7 post engraftment as compared with ^Emp^MSCs. Basal DMEM injected animal hearts showed no detection of *sry*-gene expression and served as a negative control. (B) FISH using rat y-chromosome specific probe labeled with red fluorescence to visualize transplanted male ^Shh^MSCs on day-7 post-engraftment in group-3.

Myocardial infarction model was developed in female Fisher-344 rats by permanent ligation of the coronary artery. Transplantation of the cells was carried out 10 days after the development of myocardial infarction model in order to allow local inflammatory response to subside in the infarcted heart. All animals survived full length of studies and there were no deaths related with cell transplantation. Four animals/group were harvested for molecular studies on day-4 after their respective treatment. Real-time PCR for *sry*-gene showed that the transplanted male donor cells survived significantly higher in^ Shh^MSCs transplanted animal hearts (group-3) as compared with ^Emp^MSCs transplanted animal hearts (group-2; *p*<0.01) (Figure 4). No *sry*-gene signals were observed in basal DMEM injected animal hearts (group-1) which served as a negative control. The presence of surviving male donor ^Shh^MSCs were visualized by FISH staining using fluorescently labeled rat y-chromosome specific probe (Figure 4B).

### ^Shh^MSCs Attenuated Infarction Size and Enhanced Angiogenic Potential

**Figure 5:**
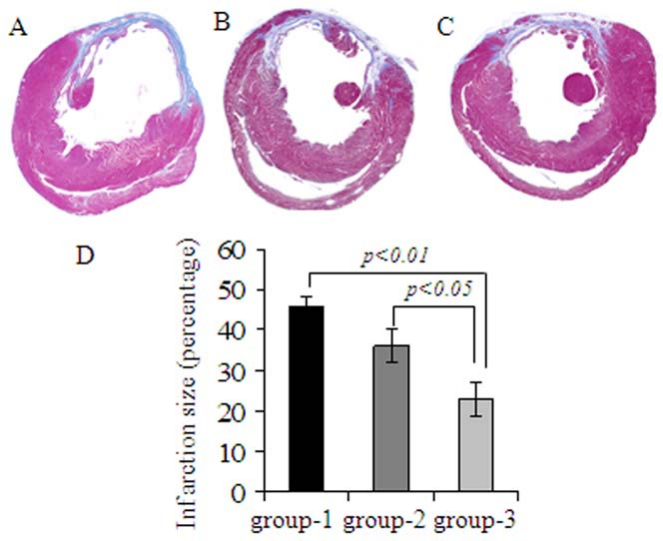
Shh overexpression attenuated infarction size expansion. Masson's trichome staining of formalin fixed paraffin embedded of the histological sections from (A) group-1 (B) group-2 and (C) group-3 animal hearts was carried out to visualize the area of fibrosis. Infarction size was significantly attenuated in group-3 after ^Shh^MSCs engraftment as compared with groups-1 and 2.

**Figure 6:**
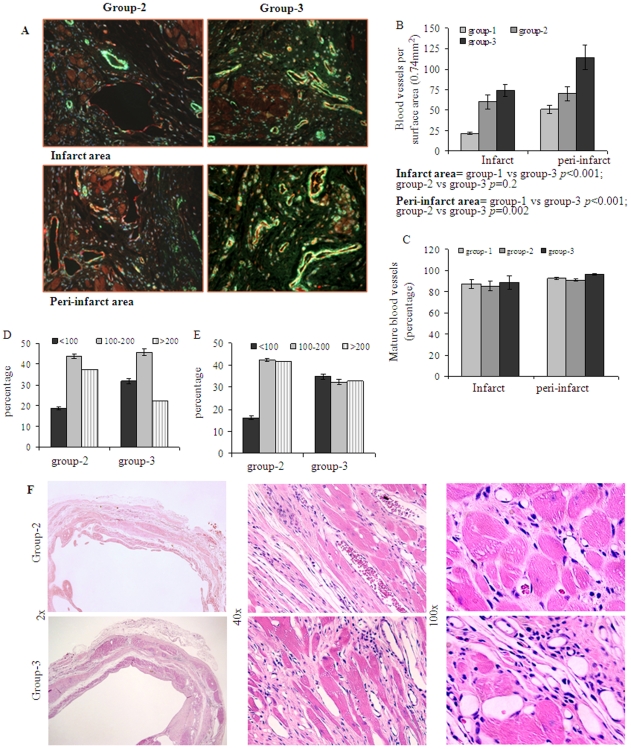
^Shh^MSCs improved blood vessel density in the infarcted heart. (A–B) Blood vessel density analysis for myocardial angiogenesis at 8-weeks after respective treatment in different groups of animals. The histological sections were immunostained for vWFactor-VIII (red) and smooth muscle actin (green) for visualization of blood vessels. The number of blood vessels per surface area (0.74 mm^2^) was significantly higher in the infarct and peri-infarct areas in group-3 (*p*<0.05) as compared with group-1 and group-2. (C) The percentage of mature blood vessels (indicated by double fluorescent immunostaining for vWFactor-VIII and smooth muscle actin) showed no significant difference between the three treatment groups. However, (D–E) showed that average size of blood vessel diameter (based on number of pixels as arbitrary unit) was more uniform in peri-infarct region of group-3. Blood vessels in the infarct and peri-infarct areas with diameter of <100 pixels (33%), 100–200 pixels (39%) and >200 pixels (27%) diameter was observed in group-3 as compared with group-2 <100 (17%), 100–200 (43%) and >200 (39%). (F) Photomicrographs of hematoxylin-eosin stained histological sections at 8-weeks after their respective treatment in group-3 and group-2. Red blood cells could be seen in some of the blood vessels as indicated by green arrows showing the functional status of the blood vessels.

**Figure 7:**
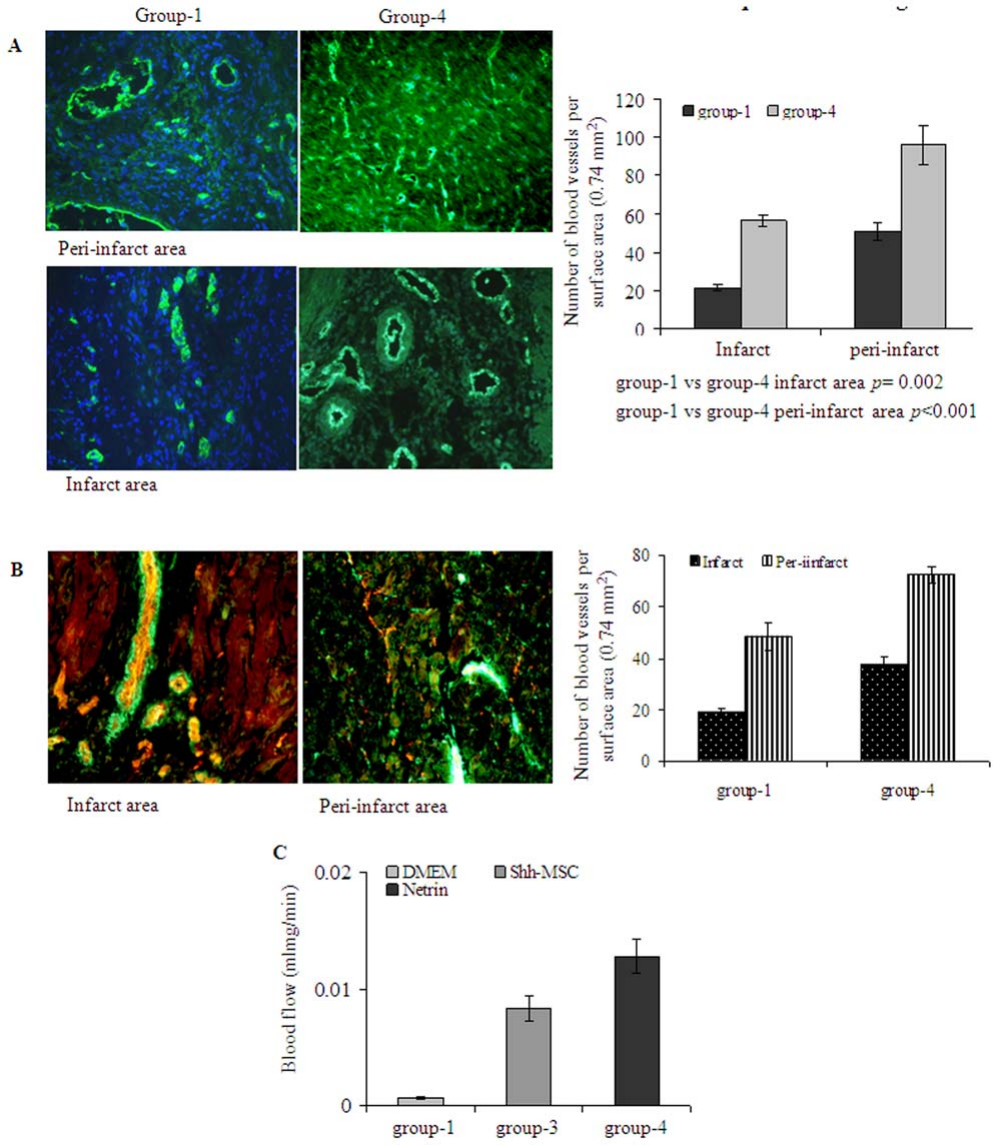
Recombinant netrin-1 treatment improved blood vessel density in the infarcted heart. (A) At 8-weeks after recombinant netrin-1 delivery to the heart (group-4), the number of blood vessels per surface area (0.74 mm^2^) was significantly higher as compared with group-1 in both infarct as well as peri-infarct regions. (B) Double fluorescent immunostaining for vWFactor-VIII (red) and smooth muscle actin (green) showed that like Shh overexpression in the heart, netrin-1 protein delivery resulted in increased arteriolar density (blood vessels double positive for vWFactor-VIII and smooth muscle actin) in group-4. (C) Functional status of blood vessels in the infarcted heart was determined by fluorescent microsphere method for regional blood flow studies assessment. Regional blood flow was significantly improved in group-3 animal hearts as compared with group-1. However, regional blood flow changed insignificantly as compared with that of normal un-infarcted heart.

Eight weeks after their respective treatment, infarction size was attenuated in group-2 and group-3 as compared with group-1 (n = 4 per group). However, only group-3 showed significant attenuation of infarction size (23±3.9%) in comparison with group-1 (45.5±2.8%; *p* = 0.01) and group-2 (36±4.01%; *p*<0.05) (Figure 5). The vascular structures in the infarcted myocardium (n = 4 per group) were visualized by fluorescence immunostaining specific for von Willebrand Factor-VIII (vWFactor-VIII) which showed significantly higher blood vessel density (number of capillaries/0.74 mm^2^) in both infarct and peri-infarct regions in group-3 in comparison with group-1 and group-2 (Figure 6A-B). Capillary density in group-3 was 74±7 and 114±15 in the infarct and peri-infarct areas respectively as compared to group-2 (60±8.8 *p* = 0.2 and 70±8.6; *p* = 0.002) and group-1 (21.5±1.5; *p*<0.001 and 50.7±4.7; *p*<0.001). Between group-3 and group-2, blood vessel density changed insignificantly in the infarct region (*p* = 0.2) but the change was significant in the peri-infarct region (*p* = 0.002). Counter immunostaining with anti-vascular smooth muscle actin for arteriolar density (the number of arterioles/0.74 mm^2^) analysis showed higher blood vessel maturation (staining positively for both vWF-VIII and vascular smooth muscle actin) in group-3 (Figure 6A–B). The percentage of mature blood vessels in infarct and peri-infarct areas was 88.8±6.1 and 96.5±0.7 in group-3 as compared to 85.4±4.6 and 91±1 in group-2 and 87.2±4.4 and 92.6±1.2 in group-1 (Figure 6C). Although the percentage of the mature blood vessels did not show any significant difference between the three treatment groups, the total number of mature blood vessels was highest in group-3, thus indicating a progressive maturation of the newly formed capillary network in the presence of Shh overexpression. Vascular diameters were also different between the three groups in the infarct (Figure 6D) and peri-infarct (Figure 6E) areas. Setting up the number of pixels as arbitrary units to determine blood vessel diameter, the percentage of blood vessels with <100 pixels was significantly smaller in group-2 in both infarct (18.7%) and peri-infarct (16.1%) areas as compared with infarct (31.9%) and peri-infarct (34.8%) in group-3 (Figure 6D). On the other hand, the percentage of medium sized blood vessels showed insignificant difference in infarct areas of group-2 (43.7%) and group-3 (45.7%) (Figure 6E). In total, (sum of blood vessels in infarct and peri-infarct areas) blood vessels with a diameter ranging from <100 pixels (33%), 100–200 pixels (39%) and >200 pixels (27%) were observed in group-3 as compared with group-2 <100 (17%), 100–200 (43%) and >200 (39%). These results indicated that ^Shh^MSCs were more efficient in induction of mature and medium sized blood vessels as compared with ^Emp^MSCs. More importantly, despite extensive neovascularization observed in our experiments, we did not witness the formation of hemangioma-like structures subsequent to engraftment of ^Shh^MSCs. Structure elucidation by hematoxylin/eosin staining revealed extensive neovascularization with peculiar vascular lacunae filled with red blood cells both in the infarct and peri-infarct regions (Figure 6F). Similar angiogenic response in the infarcted heart was achieved by injection of recombinant netrin-1. Capillary density at 40x magnification (0.74 mm^2^) in recombinant netrin-1 treated animal hearts (group-4) was 60±5.9 in infarct and 96.6±23.3 in peri-infarct areas which was significantly higher as compared with group-1 in the infarct (*p = 0.002*) and peri-infarct areas (*p<0.001*) (Figure 7A). Similarly, arteriolar density was also higher in recombinant netrin-1 treated animal hearts (group-4) in the infarct and peri-infarct areas was 37.5±3.1 and 72.5±3 in group-4 respectively as compared with group-1 (*p<0.05*) and group-2 (*p<0.05*) (Figure 7B).

### ^Shh^MSCs Improved Regional Blood Flow in the Infarcted Heart

To investigate whether vascular density in the infarct and peri-infarct regions were paralleled by increased regional blood flow, myocardial perfusion analysis was performed using fluorescent microspheres (n = 3 per group; Figure 7C). Blood perfusion in the infarcted myocardium was restored after implantation of ^Shh^MSCs (group-3) in comparison with DMEM injected animals (group-1). The average blood flow quantified in the infarcted heart in group-3 was 0.01±0.001 ml/mg/min which was significantly improved as compared to group-1 0.0002 ml/mg/min. Although this represented a significant 100-fold increase in the myocardial regional blood flow in comparison with group-1, this increase in group-3 was insignificant as compared with the normal heart (0.02±0.001 ml/mg/min).

### ^Shh^MSCs Improved Heart Function

**Figure 8:**
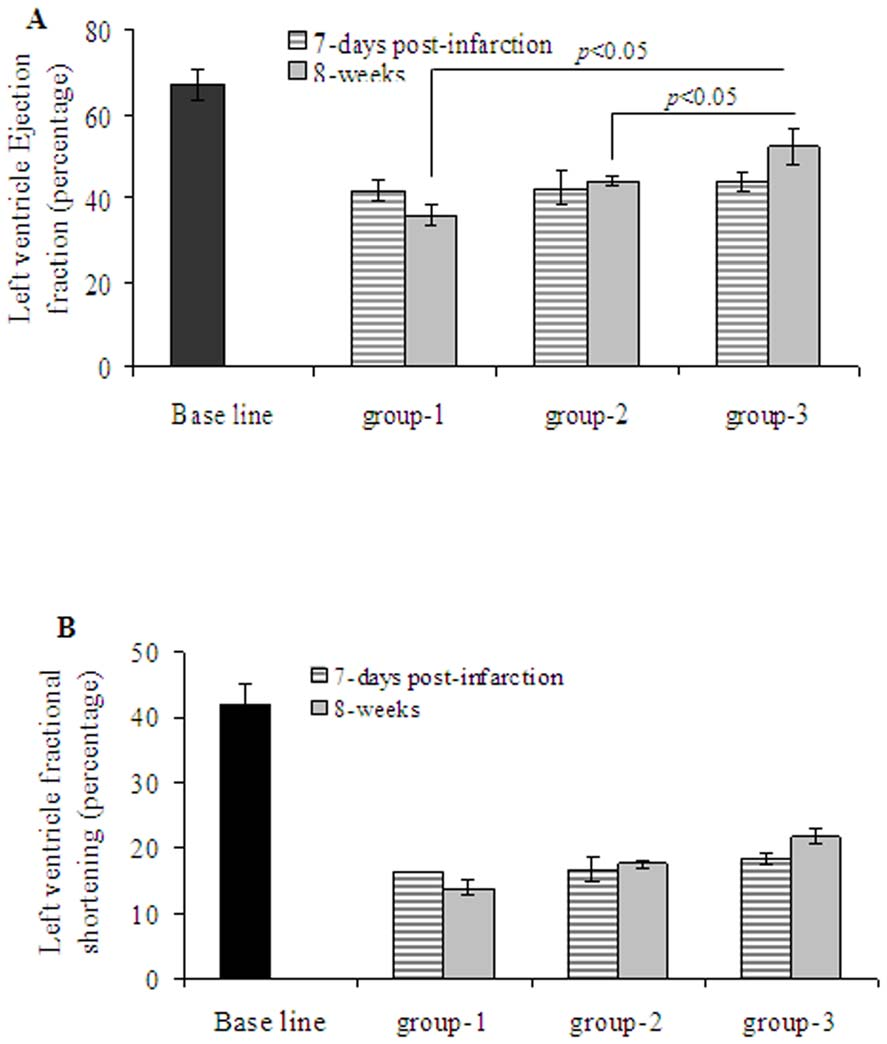
^Shh^MSCs transplantation preserved global function of the infarcted heart. Echocardiographic assessment of the heart function indices (LVEF and LVFS) showed that preservation of the global heart function at 8 weeks after their respective treatment was significantly better in group-3 in comparison with DMEM injected group-1 and ^Emp^MSCs transplanted group-2. LVEF and LVFS were calculated 1-week after myocardial infarction (before their respective treatment) and 8-weeks after respective treatment. Results are shown as mean±SEM (n = 7 animal per group).

Assessment of LV contractile and remodeling indices was performed on day-7 after myocardial infarction (one day prior to cell transplantation) and 8 weeks after cell transplantation (n = 7 per group). Echocardiography showed deterioration of indices of LV contractile function in all the three groups at 7-days after myocardial infarction without any significant difference (*p*>0.05 between all the groups) (Figure 8). Thus, at 7-days post-infarction, LV ejection fraction (LVEF) and LV fractional shortening (LVFS) in group-1 were (41.9±2.55% and 16.3±1.1), group-2 (42.3±4.4% and 16.7±1.8) and group-3 (43.9±2.2% and 18.3±0.8) as compared with the baseline (67.3±3.4 and 42±3) respectively. At 8-weeks after their respective treatment, we observed significantly deteriorated LVEF (35.8±2.3%) and LVFS (13.8±1.17%) in group-1 (Figure 8A). On the contrary, there was significant attenuation of LVEF and LVFS in group-2 (44.1±1.2%; 17.6±0.6%) and group-3 (52.3±4.4%; 21.8±1.2%) as compared to the corresponding values of baseline echocardiography (67.3±3.4%; 21.8±1.2%). As a marker of LV remodeling, LV end diastolic diameter (LVEDD) and LV end systolic diameter (LVESD), anterior wall thickness and posterior wall thickness were assessed as shown in Table S4. Echocardiography performed before and after their respective treatment in the experimental and control groups clearly demonstrated that intramyocardial administration of ^Shh^MSCs had favorable impact on preservation of LV function in rats with chronic myocardial infarction.

## Discussion

We have shown that in vitro reprogramming of MSCs maximizes their survival and angiogenic potential in the infarcted heart. The salient findings of the study are: 1- Genetic modification of MSCs with Shh transgene results in multifold increased iNOS upregulation and NO production, an effect which was associated with higher level of angiogenic growth factor expression including VEGF, Ang-1 and netrin-1. 2- The induction of iNOS in ^Shh^MSCs occurred in PKC dependent manner. 3- ^Shh^CM mediated increased angiogenic response was abrogated in the presence of Shh and netrin-1 specific antibodies. 4- Intramyocardial engraftment of ^Shh^MSC induced significant angiogenesis, improved regional blood flow and significantly preserved global heart function. We propose that the increased angiogenesis is due to the production of NO with simultaneous upregulation of multiple angio-competent factors including netrin-1. The results from the present study also indicated that genetically engineered ^Shh^MSCs promoted migration of endothelial progenitor cells which may be attributed to the dramatic upregulation of MMP-9 (4-fold) in ^Shh^MSCs.

Sonic hedgehog (Shh) together with Desert hedgehog (Dhh) and Indian hedgehog (Ihh) are three members of the hedgehog gene family identified in the mammals [15]. Hedgehog signaling involves binding of hedgehog with its ptch-1 receptor which in turn releases its inhibitory effect on Smoothened (*smo*). Activated *smo* then initiates signaling events which lead to regulation of transcriptional factors belonging to the *Gli* family and its relevant downstream genes [16]. Shh is a secretary protein and therefore Shh gene delivery has been assessed in experimental animal models of the infarcted heart [6]. Results from these studies indicate that postnatal reconstitution of Shh signaling resulted in tissue preservation and repair, and that gene therapy with Shh not only prevented fibrosis, it also promoted angiogenesis and regional blood flow. Enhanced angiogenesis may be attributed to the multiple effects of localized Shh transgene expression. Activation of Shh pathway upregulates the expression of multiple angiogenic cytokines, including VEGF, angiopoietins, SDF-1α and IGF-1 and development of capillary network [4], [17]. Our results were in harmony with these data and further showed uniquely that Shh gene overexpression up-regulated iNOS, netrin-1 and HGF in addition to the already reported cytokines. HGF is a mitogen of mesenchymal origin, and is also reported to stimulate NO production and endothelial cell motility through upregulation of iNOS [18]. Put together, these molecular changes lead to a significant increase in biologically active NO production in ^Shh^MSCs.

iNOS is expressed following inflammatory or growth factor stimulation of cells unlike its counterpart eNOS (endothelial nitric oxide synthase) and nNOS (neuronal nitric oxide synthase) which are constitutively expressed. Activation of iNOS can produce copious amounts of NO for longer duration [19]. Generated from the NOS enzyme activity, NO is recognized as an important regulator of cardiovascular system functionality. Whereas NO inhibits proliferation of smooth muscle cells, gene delivery of iNOS to endothelial cells is protective for the cells via NO release without an influence on their proliferation [20]. Secondly, endothelial cell migration is an essential component of several vascular processes including the maintenance of endothelial integrity and angiogenesis. It is suggested that endothelial cells must exhibit a phenotype of non-directional motility as a prerequisite for responding to stimuli for migration. The same study proposes that NO plays an obligatory role in eliciting this phenotype. More recent studies have shown that NO generated from iNOS activity modulates the expression of matrix metalloproteinase-9 (MMP-9), an enzyme responsible for degradation of extracellular matrix, which significantly influenced cell migration [21]. A few previous studies have reported increased MMP-9 the Shh-expressing cells, which was attenuated by the inhibition of EGF receptor activation or blocking the EGF receptor and ligand interaction [22]. In doing so, Shh has been shown to directly stimulate EGFR signaling. In the present study, prior treatment of cells with cyclopamine and chel abrogated the expression of iNOS and angiogenic growth factors including VEGF, Ang-1 and netrin-1 thus suggesting that these pro-angiogenesis relevant molecular events were PKC dependent.

Netrins are important in axonal guidance, regulation and maintenance of central nervous system and are also involved in the development of mammary gland, lung, pancreas, and blood vessel [23]. Even though there is some controversy, most recent studies suggested that netrin-1 functions as a pro-angiogenic factor. In vivo studies also indicated that netrins promoted neovascularization and reperfusion in a murine model of peripheral vascular disease and also induced migration, proliferation and tube formation during in vitro studies involving multiple endothelial cell lines [24]. Characterizing a mechanism for netrin-1 induced angiogenesis, a critical role for NO has been elucidated subsequent to feed-forward ERK1/2 and eNOS activation in endothelial cells treated with netrin-1 [25]. Our results showed that netrin-1 expression increased significantly in ^Shh^MSCs both at the protein and RNA levels in PKC (PKM) dependent fashion.

Another interesting finding in the present study was the presence of PKM fragment of PKC in ^Shh^MSCs at 72-h after transfection with Shh plasmid. PKM is a constitutively active 40-kDa catalytic fragment of PKC [26]. The regulatory role of PKC in Shh signaling has been reported previously [22]. In a study involving NIH 3T3 cells, PKC-δ was integral to hedgehog signaling to promote proliferative activity of the cells [27]. In our present study, treatment of ^Shh^MSCs with both the PKC inhibitor chel and Shh inhibitor cyclopamine abolished PKM fragment which indicated that PKM was downstream of Shh and that PKC was essential for its upregulation. White et al. (2007) while experimenting on intact sea urchin spermatozoa showed PKC, most likely through its cleavage into active catalytic product PKM, was the central signaling mediator associated with maintenance of sperm mobility [26]. PKC inhibitors such as chel and calphostin-C, as well as staurosporine, were found to rapidly arrest the motility of sea urchin spermatozoa freshly released into seawater. At the same time, these inhibitors prevented the motility-associated increase in phosphorylation of several PKC substrates. Pretreatment of ^Shh^MSCs with these inhibitors abrogated PKM with concurrent abrogation of iNOS and netrin-1.

Another novel finding of our study supported by molecular and histological data was the improved survival of ^Shh^MSCs post engraftment. In view of the reported data that massive loss of the transplanted cells occurs after transplantation remains a major determinant of the effectiveness of heart cell therapy, ^Shh^MSCs are at an advantage in terms of their survival after engraftment. Histological studies at eight weeks after cell transplantation showed a marked reduction in infarct size and preservation of host myocardium in ^Shh^MSCs transplanted group as compared with ^Emp^MSC and DMEM groups. The cytoprotective effects were accompanied by a significant increase in capillary density and a higher number of mature blood vessels (smooth muscle actin positive cells) in ^Shh^MSCs group. These results are consistent with earlier studies using direct injection of Shh plasmid into the infarcted heart [4], [6], [24].

In conclusion ^Shh^MSCs showed upregulation of several angiogenic cytokines and signaling molecules including Ang-1, VEGF, IGF, HGF, iNOS and netrin-1. Additionally, ^Shh^MSCs also generated NO at significantly higher levels. These molecular changes were mediated by iNOS/netrin/PKC signaling pathway downstream of Shh gene overexpression which combined with stem cell transplantation could be a promising strategy for the treatment of an infarcted heart.

## Supporting Information

Text S1 (0.06 MB DOC)

Figure S1 Construction of Shh plasmid. (A) Vector Map used in construction of Shh-plasmid. The vector backbone was purchased from commercial source (Stratagene, USA) and Shh mRNA was isolated from 14-day rat embryo, used for cDNA synthesis and cloned into pCMV Script vector. (B) Sequence of pCMV Shh-vector using T7 and T3 primers showing sequence of Shh gene insert. (C) Flow cytometry for surface marker expression showed that overexpression of Shh transgene did not alter the expression of surface markers in ^Shh^MSCs (indicated by red line) including CD44, CD59, CD105 and CD106 as compared with the Empty vector transfected MSCs (^EMP^MSCs; indicated by black line).
(2.61 MB TIF)

Figure S2 Abrogation of PI3K in ^Shh^MSCs using PI3K specific siRNA. Western blot showing successful abrogation of PI3K in ^Shh^MSCs following transfection with PI3K specific siRNA. ^Shh^MSCs transfected with scrambled siRNA (Sc siRNA) and ^Emp^MSCs with siRNA transfection were used as controls. Successful abrogation of PI3K was indicated by loss of Akt phosphorylation.
(0.85 MB TIF)

Figure S3 Cytoprotective effects of conditioned medium from ^Shh^MSCs (^Shh^CM) on native MSCs. LDH release assay showed that ^Shh^CM was significantly more protective for native MSCs against oxidant stress as compared with conditioned medium from ^Emp^MSCs (^Emp^CM).
(1.05 MB TIF)

Figure S4 Cytoprotective effects of conditioned medium from ^Shh^MSCs (^Shh^CM) on native H2C9 cardiomyocytes. LDH release assay showed that ShhCM was significantly more protective for H2C9 cardiomyocytes against oxidant stress as compared with conditioned medium from ^Emp^MSCs (^Emp^CM).
(1.17 MB TIF)

Table S1 Primary antibodies used for Western immunoblotting and immunohistochemistry.
(0.03 MB DOC)

Table S2 Primers used for classic and real-time PCR.
(0.03 MB DOC)

Table S3 Fold change in different growth factor and cytokine expression in ^Shh^MSCs as compared with ^Emp^MSCs.
(0.03 MB DOC)

Table S4 The heart function indices measured by echocardiography on (A) day-7 and (B) 8-weeks after cell transplantation.
(0.03 MB DOC)
