# Supplementary material for: Sonic Hedgehog Gene Delivery to the Rodent Heart Promotes Angiogenesis via iNOS/Netrin-1/PKC Pathway
Source: PLoS One. 2010 Jan 5;5(1):e8576. doi: 10.1371/journal.pone.0008576 (PMC2797399; doi:10.1371/journal.pone.0008576)
Supplement: Text S1 — (0.06 MB DOC) [file pone.0008576.s001.doc]

**Supporting Information**

**Materials and Methods**

Purified mouse netrin-1 was purchased from R & D systems. The antibodies used for Immunostaining and Western blotting and the primer sequences used for RT-PCR given in Supplementary Table-1 and Supplementary Table-2 respectively. Scrambled siRNA (Sc siRNA), PI3K siRNA and lipofectamine for transfection were purchased from Qiagen. Fugene HD transfection reagent was purchased from Roche. Cyclopamine was purchased from Calbiochem.

**Purification and propagation of MSCs**

MSCs were isolated from young male Fischer-344 rats as previously described [Supporting reference 1].

**Shh plasmid construction and transfection**

For construction of Shh plasmid, 603-bp amino terminal domain coding sequence of rat Shh was cloned into pCMV-ScriptPCR mammalian expression vector (Stratagene). Expression of Shh transgene was driven by a cytomegalovirus promoter sequence and downstream from the Shh cDNA was an SV40 polyadenylation sequence. Additionally, the plasmid contained a gene that conferred neomycin/kanamycin resistance to the host cells. Transfection of Shh plasmid into MSCs was carried out using FuGENE HD transfection reagent with different ratios of plasmid and transfection reagent (Roche) according to the manufacturer’s directions. For optimal transfection efficiency, 5x10^5^  cells were seeded in a 10cm culture dish 24 h prior to transfection. Transfection complex of Fugene and Shh plasmid DNA was prepared using 3: 2 ratio in serum free medium and added to the cells per instructions of the manufacturer. Transfection was allowed for 8-12 h before the removal of transfection complex. Transfection efficiency was assessed by immunostaining and real-time PCR. The transfected MSCs (^Shh^MSCs) were characterized for surface marker expression by flow cytometry using our standard protocol [Supporting reference 1]. For collection of the conditioned medium, the transfected cells were cultures for 72 h in 0.5% serum containing cell culture medium. The medium thus collected was centrifuged to remove any cell debris before use in further experiments.

**In vitro migration assay**

For migration assay, HUVECs were seeded at a density of 5x10^4^ cells per well onto 8-µm Transwell inserts (Transwell Coster Corning Inc.). The lower chamber was filled with 500μl low serum cell culture media containing the test factors. The cells were incubated for 4 h at 37ºC (5%CO_2_). Migration activity was evaluated as the mean number of migrated cells in 3 high power fields (100x) per well. The experiment was performed in triplicate.

**In vitro tube formation assay**

Matrigel (60ul, BD Bioscience) was applied to each well of a 96-well plate and incubated at 37ºC for 30 min before adding 12.5x 10^3^ HUVECs per well in serum free media. After 45 min at 37ºC and 5%CO_2_, serum free media was removed and replaced with conditioned media from ^Shh^MSCs (^Shh^CM) and ^Emp^MSCs (^Emp^MSCs). Tube formation was assessed at 4 h after their respective treatment in triplicate. Phase contrast photomicrographs of representative fields were taken at 20x magnification and counted for branch points per microscopic field.

**Western blotting**

MSCs were transfected with control (empty vector) or Shh plasmid using Fugene HD transfection reagent in growth media containing 10%FBS without antibiotics. For studies using PKC inhibitor, the cells were treated with 2.5 μM chel for 30 min before Shh gene transfection. For siRNA studies, the cells were co-transfected with PI3K specific siRNA and Shh plasmid. Control cells were co-transfected with scrambled siRNA and Shh plasmid. The cells were harvested after 72 h and Western blot analysis was performed as previously described [Supporting reference 2]. Western blot analysis for various proteins using their respective antibodies [Supporting Table S1].

**Reverse transcription polymerase chain reaction**

Isolation of total RNA from the different groups of MSCs, and their subsequent first-strand cDNA synthesis, was performed using an RNeasy mini kit (Qiagen) and an Omniscript Reverse Transcription kit (Qiagen), respectively, per the instructions of manufacturer. Real-time PCR was performed using iQ SYBR-Green supermix (Bio-Rad) in a Bio-Rad iQ5 optical module [Supporting reference 3]. The final concentration of MgCl_2_ used in the PCR mix was 3.5 mM. The cycling conditions were set at 3 min at 95°C for initial denaturation, 40 cycles of denaturation at 95°C for 30 sec, annealing at 59.5°C for 40 sec and extension at 72°C for 50 sec. The data was acquired during the extension step. Melting curves were obtained at the end of the reaction by gradually raising the temperature by 1°C/min from 59.5°C to 95°C over a time period of 35 min. Conventional RT-PCR was performed as previously described [Supporting reference 3].

For *sry*-gene expression studies, gDNA was isolated from the rat heart tissue samples collected on day-7 after cell transplantation and was used for real time PCR studies [Supporting reference 3]. The primer sequences used have been shown in Supporting Table S2.

**Nitric oxide synthase activity assay**

Nitric oxide activity was determined in ^Shh^MSCs and ^Emp^MSCs using a colorimetric NO assay kit from Oxford Biomedical Research. NADPH and L-arginine are required for the continual operation of NOS and production of nitric oxide (NO). To measure iNOS activity, samples were incubated with NADPH for 100 min which facilitate NO production. NO rapidly degrades to nitrate and nitrite. Nitrate is converted to nitrite by using recombinant nitrate reductase, which was followed by spectrophotometric quantification of nitrate using Griess Reagent per instructions of the manufactures.

**Cytoprotective effect of ^Shh^CM and LDH release assay.** The cytoprotective effects of ^Shh^CM were determined as previously described [Supporting reference 4].

**In vivo studies**

**Experimental animal model and cell engraftment**

The present study conformed to the Guide for the Care and Use of Laboratory Animals published by the US National Institutes of Health (NIH Publication No. 85-23, revised 1985) and protocol approved by the Institutional Animal Care and Use Committee, University of Cincinnati.

A model of chronic myocardial infarction (MI) was developed in young female Fischer-344 rats each weighing 180-200g as described previously [Supporting reference 1]. Following tracheal intubation and ventilation using Harvard Rodent Ventilator (Model-683), the hearts were exposed. Experimental MI was created by permanent ligation of the left anterior descending coronary artery. Eight days later the animals were grouped to receive intra-myocardial injections of 70µl of basal DMEM without cells (group-1) or containing 1x10^6^ ^Emp^MSCs (group-2), and 1x10^6 Shh^MSCs (group-3). In group-4, the animals received one time administration of 500ng recombinant netrin-1 protein in 50μl of basal DMEM. Each animal received multiple injections (n=4 on average) in the centre and border zone of the infarct using a 27G needle on beating heart. The chest of the animal was closed and the animal was allowed to recover. The animals were maintained on Buprinex after surgery for 24 h to alleviate pain.

**Assessment of heart function by Echocardiography**

Transthoracic echocardiography was performed to study change in the heart function before cell transplantation (7 days after MI) and 8 weeks after respective treatment using Compact Linear Array probe CL10-5 on HDI-5000 SONOS CT (HP) as described earlier [Supplementary reference 1]. Anterior and posterior end-diastolic and end-systolic wall thickness and left ventricle (LV) internal dimensions; LV end-systolic (LVESD) and end-diastolic (LVEDD) diameters were measured from at least three consecutive cardiac cycles. Indices of LV systolic functions including LV fractional shortening (LVFS) and LV ejection fraction (LVEF) were calculated using LVFS= (LVEDD-LVESD)/LVEDd x100 and LVEF= [(LVEDD^3^-LVESD^3^)/ LVEDD^3^] x100 relations respectively and the results were expressed as percentage.

**Histochemical and Immunohistochemical studies**

For measurement of infarction size and area of fibrosis, the hearts were arrested in diastole by intravenous injection of cadmium chloride and fixed in 10% buffered formalin. The heart was then excised, cut transversely, and embedded in paraffin. Histological sections of 6µm thickness were cut and used for hematoxylin-eosin and Masson’s trichome staining for visualization of muscle architecture and area of fibrosis. Infarct size was defined as sum of the epicardial and endocardial infarct circumference divided by sum of the total LV epicardial and endocardial circumferences using computer-based planimetry with Image-J analysis software (version 1.6065; NIH) [Supporting reference 1].

Fluorescent *in situ* hybridization (FISH) on the histological sections was performed using rat 12/Y-chromosome paint labeled with Cy3 fluorescent label (cat# CA1631) per instructions of the manufacturer.

Blood vessel density was assessed as previously described [Supporting reference 2]. Briefly, cryosections (6µm thick) were immunostained using vonWillebrand Factor-VIII (vWF) specific primary antibody (1:50; Dako, Denmark) and detected with fluorescently labeled secondary antibody (Molecular Probes). The number of blood vessels positive for vWF-VIII were counted in both infarct and peri-infarct regions. At least 50 microscopic fields each in infarct and peri-infarct regions were randomly selected and counted in each treatment group**.** Blood vessel density was expressed as the number of vessels per microscopic surface area (0.74mm^2^) at 200x magnification. Blood vessel maturation index was determined by co-immunostaining with both vWF-VIII and Smooth muscle actin (SMA) and counting the number of vessels positive for both vWF-VIII and SMA. Blood vessel diameter was calculated by measuring the circumference of the blood vessels. Based on the diameter the blood vessels were divided into 3 categories, diameter > 100 pixels, 100-200 pixels and < 200 pixels.

**Statistical analysis**

All data were described as mean ±SEM. To analyze the data statistically, we performed Student’s t-test and one-way ANOVA with post hoc analysis and a value of *p*<0.05 was considered statistically significant.

**Supporting References**

1. Jiang S, Haider H, Idris NM, Salim A, Ashraf M (2006) Supportive interaction between cell survival signaling and angiocompetent factors enhances donor cell survival and promotes angiomyogenesis for cardiac repair. Circ Res 99:776-84.

2. Niagara MI, Haider H, Jiang S, Ashraf M (2007) Pharmacologically preconditioned skeletal myoblasts are resistant to oxidative stress and promote angiomyogenesis via release of paracrine factors in the infarcted heart. Circ Res 100:545-55.

3. Shujia J, Haider HK, Idris NM, Lu G, Ashraf M (2008) Stable therapeutic effects of mesenchymal stem cell-based multiple gene delivery for cardiac repair. Cardiovasc Res 77:525-33.

4. Haider HKh, Jiang S, Idris NM, Ashraf M (2008) [IGF-1-overexpressing mesenchymal stem cells accelerate bone marrow stem cell mobilization via paracrine activation of SDF-1alpha/CXCR4 signaling to promote myocardial repair.](http://www.ncbi.nlm.nih.gov/pubmed/18948617?ordinalpos=4&itool=EntrezSystem2.PEntrez.Pubmed.Pubmed_ResultsPanel.Pubmed_DefaultReportPanel.Pubmed_RVDocSum) Circ Res 103:1300-08.
