# Supplementary figures and images for: Sonic Hedgehog Gene Delivery to the Rodent Heart Promotes Angiogenesis via iNOS/Netrin-1/PKC Pathway
Source: PLoS One. 2010 Jan 5;5(1):e8576. doi: 10.1371/journal.pone.0008576 (PMC2797399; doi:10.1371/journal.pone.0008576)

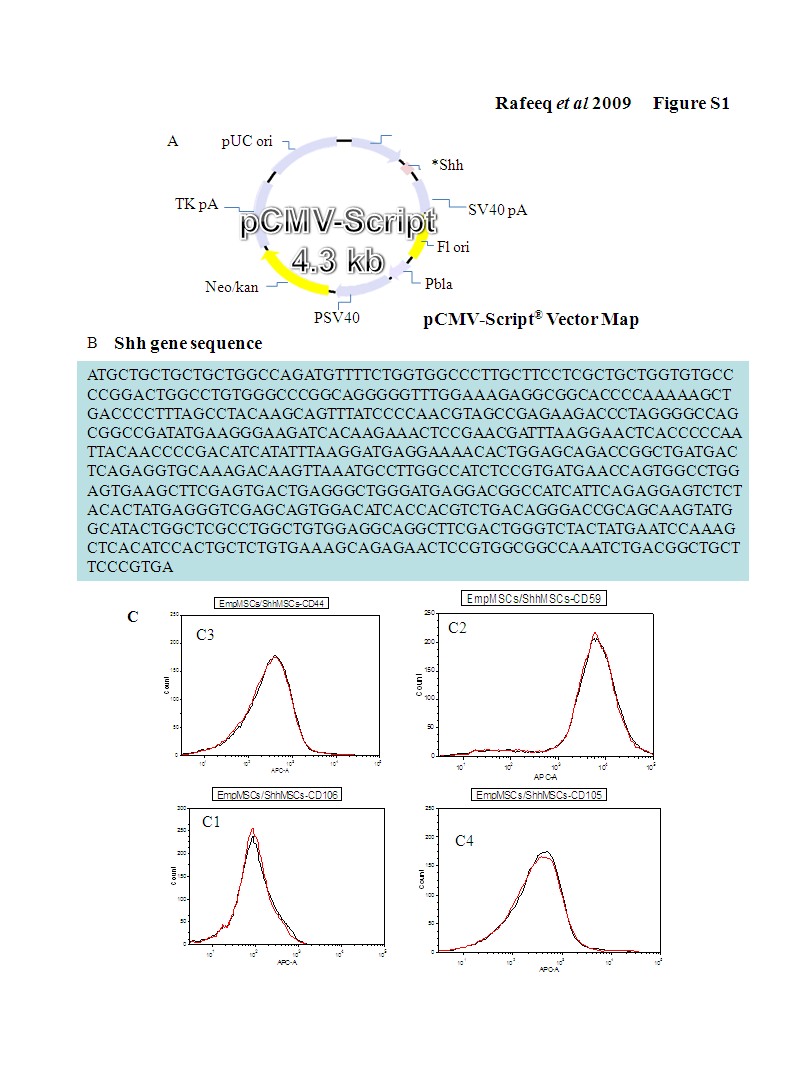

Supplement: Figure S1 — Construction of Shh plasmid. (A) Vector Map used in construction of Shh-plasmid. The vector backbone was purchased from commercial source (Stratagene, USA) and Shh mRNA was isolated from 14-day rat embryo, used for cDNA synthesis and cloned into pCMV Script vector. (B) Sequence of pCMV Shh-vector using T7 and T3 primers showing sequence of Shh gene insert. (C) Flow cytometry for surface marker expression showed that overexpression of Shh transgene did not alter the expression of surface markers in ShhMSCs (indicated by red line) including CD44, CD59, CD105 and CD106 as compared with the Empty vector transfected MSCs (EMPMSCs; indicated by black line). (2.61 MB TIF) [file pone.0008576.s002.tif]

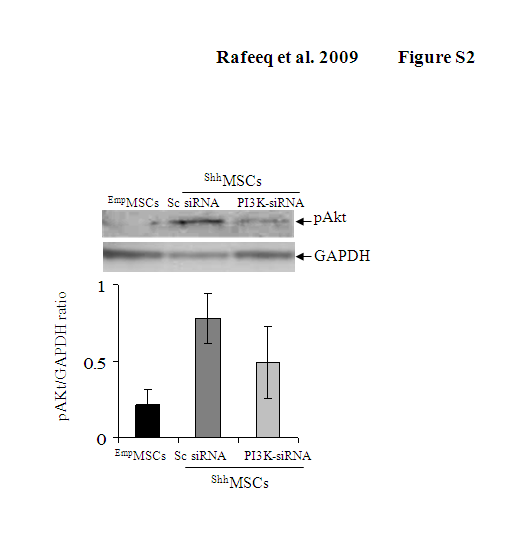

Supplement: Figure S2 — Abrogation of PI3K in ShhMSCs using PI3K specific siRNA. Western blot showing successful abrogation of PI3K in ShhMSCs following transfection with PI3K specific siRNA. ShhMSCs transfected with scrambled siRNA (Sc siRNA) and EmpMSCs with siRNA transfection were used as controls. Successful abrogation of PI3K was indicated by loss of Akt phosphorylation. (0.85 MB TIF) [file pone.0008576.s003.tif]

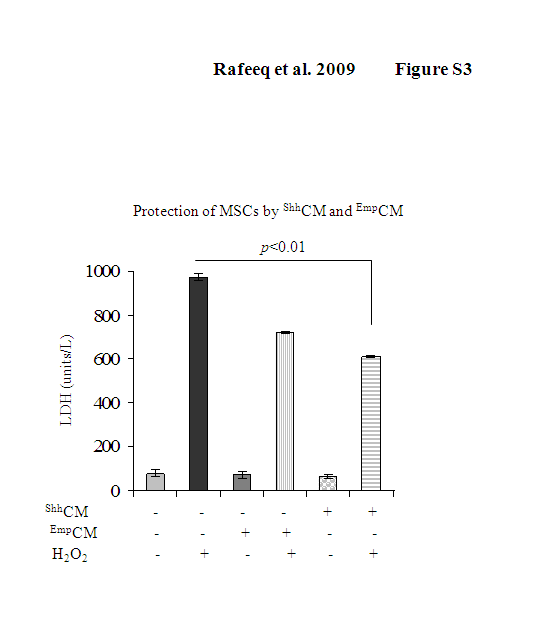

Supplement: Figure S3 — Cytoprotective effects of conditioned medium from ShhMSCs (ShhCM) on native MSCs. LDH release assay showed that ShhCM was significantly more protective for native MSCs against oxidant stress as compared with conditioned medium from EmpMSCs (EmpCM). (1.05 MB TIF) [file pone.0008576.s004.tif]

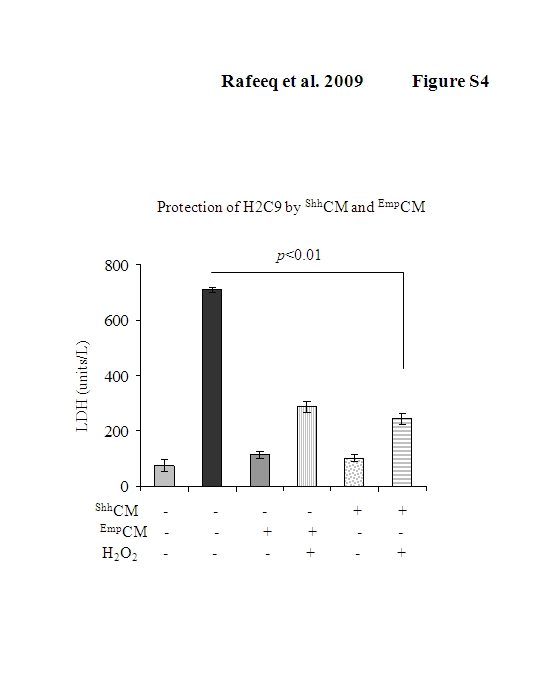

Supplement: Figure S4 — Cytoprotective effects of conditioned medium from ShhMSCs (ShhCM) on native H2C9 cardiomyocytes. LDH release assay showed that ShhCM was significantly more protective for H2C9 cardiomyocytes against oxidant stress as compared with conditioned medium from EmpMSCs (EmpCM). (1.17 MB TIF) [file pone.0008576.s005.tif]
