# Supplementary material for: Sonic Hedgehog Gene Delivery to the Rodent Heart Promotes Angiogenesis via iNOS/Netrin-1/PKC Pathway
Source: PLoS One. 2010 Jan 5;5(1):e8576. doi: 10.1371/journal.pone.0008576 (PMC2797399; doi:10.1371/journal.pone.0008576)
Supplement: Table S1 — Primary antibodies used for Western immunoblotting and immunohistochemistry. (0.03 MB DOC) [file pone.0008576.s006.doc]

**Table S1.** Primary antibodies used for Western immunoblotting and immunohistochemistry.

Antibody Dilution Source

Angiopoietin-1 1:500/WB Sigma

Actin 1:1000/WB Sigma

α-sarcomeric actinin 1:50/IM Sigma

GAPDH 1:5000/WB Cell Signaling

iNOS 1:1000/WB BD Pharmingen

Netrin-1 1:1000/WB Abcam

pAkt 1:500/WB Cell Signaling Tech.

pPKC 1:1000/WB Cell Signaling Tech.

Ptc-1 1:200/WB Santa Cruz

Shh 1:100/WB Santa Cruz

Smooth muscle actin 1:100/IM Abcam

Total Akt 1:2000/WB Cell Signaling Tech.

vWF-VIII 1:50/IM Dako

VEGF 1:1000/WB Santa Cruz

________________________________________________________________________

Abbreviations: IM= Immunostaining; WB= Western blot
