# Supplementary material for: Sonic Hedgehog Gene Delivery to the Rodent Heart Promotes Angiogenesis via iNOS/Netrin-1/PKC Pathway
Source: PLoS One. 2010 Jan 5;5(1):e8576. doi: 10.1371/journal.pone.0008576 (PMC2797399; doi:10.1371/journal.pone.0008576)
Supplement: Table S2 — Primers used for classic and real-time PCR. (0.03 MB DOC) [file pone.0008576.s007.doc]

**Table S2.** Primers used for classic and real-time PCR.

Primer Sequence Product size

Ang-1 5' cagcacaaaggacgctgata 3' 232

5' atagcgccttcagaagtcca 3'

bFGF 5' ccagttggtatgtggcactg 3' 225

5' cagggaagggtttgacaaga 3'

CD31 5' cgaaatctaggcctcagcac 3' 227

5' cgaaatctaggcctcagcac 3'

CD34 5' gggtagctctctgcctgatg 3' 204

5' tctctgagatggctggtgtg 3'

HGF 5' cgagctatcgcggtaaagac3' 165

5' tgtagctttcaccgttgcag 3'

HIF-1α 5' TCAAGTCAGCAACGTGGAAG 3' 198

5' TATCGAGGCTGTGTCGACTG 3'

IGF-1 5' tctgaggaggctggagatgt 3' 240

5' gttccgatgttttgcaggtt 3'

iNOS 5' AGGGAGTGTTGTTCCAGGTG 3' 232

5' TCCTCAACCTGCTCCTCACT 3'

MMP9 5' CAAACCCTGCGTATTTCCAT 3' 223

5' AGAGTACTGCTTGCCCAGGA 3'

Netrin-1 5' TACTGCAAGGCTTCCAAAGG 3' 193

5' AACGGATCCACAAACTCTGG 3'

Ptc1 5' ATTTCTTGCCCTTGGTGTTG 3' 166

5' GAAGGCAGTGACATTGCTGA 3'

Shh 5' AAATGCCTTGGCCATCTC 3' 243

5' TTTCACAGAGCAGTGGATGC 3'

*sry* 5’GAGGCACAAGTTGGCTCAACA3 115

5’ctcctgcAAAAAGGGCCTTT3’

VEGF 5' caatgatgaagccctggagt 3' 211

5' tttcttgcgctttcgttttt3'

Actin 5' agccatgtacgtagccatcc 3' 228

5' ctctcagctgtggtggtgaa 3'
