# Supplementary material for: Sonic Hedgehog Gene Delivery to the Rodent Heart Promotes Angiogenesis via iNOS/Netrin-1/PKC Pathway
Source: PLoS One. 2010 Jan 5;5(1):e8576. doi: 10.1371/journal.pone.0008576 (PMC2797399; doi:10.1371/journal.pone.0008576)
Supplement: Table S3 — Fold change in different growth factor and cytokine expression in ShhMSCs as compared with EmpMSCs. (0.03 MB DOC) [file pone.0008576.s008.doc]

**Table S3.** Fold change in different growth factor and cytokine expression in ^Shh^MSCs as compared with ^Emp^MSCs.

**Cytokine/growth factor Fold change**

iNOS 3436.3

Netrin-1 62.4

Angiopoietin-1 5.1

MMP-9 3.97

IL1b 7.1

HGF 6.6

VEGF 2.3

IGF-1 2.1
