# Supplementary material for: Sonic Hedgehog Gene Delivery to the Rodent Heart Promotes Angiogenesis via iNOS/Netrin-1/PKC Pathway
Source: PLoS One. 2010 Jan 5;5(1):e8576. doi: 10.1371/journal.pone.0008576 (PMC2797399; doi:10.1371/journal.pone.0008576)
Supplement: Table S4 — The heart function indices measured by echocardiography on (A) day-7 and (B) 8-weeks after cell transplantation. (0.03 MB DOC) [file pone.0008576.s009.doc]

**Table S4:** The heart function indices measured by echocardiography on (A) day-7 and (B) 8-weeks after cell transplantation.

Group-1Group-2Group-3Baseline

LVEF (%)45.2 ± 1.4 47.7 ± 4.243.95 ± 2.2 67±3.4

LVFS 18.4 ± 1.1 18.5 ± 1.818.33 ± 0.8 42±3

LVEDd(mm) 13.37 ± 0.3 13.10 ± 0.3 13.66 ± 0.2 4.4±1

AWTs(mm) 1.00 ± 0.01.01 ± 0.07 1.01 ± 0.04 2.9±0.1

**Table-SIVA**

Group-1Group-2Group-3Baseline

LVEF (%)39.61 ± 2.8 44.1 ± 1.252.3 ± 4.4 67±3.4

LVFS 16.15 ± 0.8 17.6 ± 0.621.8 ± 1.2 42±3

LVEDd(mm) 14.13 ± 1.2 15.02 ± 0.3 16.3 ± 0.5 4.4±0.9

AWTs(mm) 0.95 ± 0.110.75 ± 0.3 1.00 ± 0.18 2.9±0.1

**Table-SIVB**
